# Analyzing clinical characteristics of patients with different cumulative hemodialysis durations: a cross-sectional study

**DOI:** 10.7717/peerj.10852

**Published:** 2021-03-09

**Authors:** Xu Chen, Li Yuan, Yuan Zhang, Houyong Dai, Yaping Fan, Xiaolan Chen

**Affiliations:** Department of Nephrology, Affiliated Hospital of Nantong University, Nantong, China

**Keywords:** Clinical characteristics, Hemodialysis, Kidney failure, Anemia, Serum albumin, Age of onset

## Abstract

**Background:**

The objective of this study was to examine the clinical characteristics of patients with different cumulative hemodialysis (HD) durations, so as to improve their survival rate.

**Methods:**

In this cross-sectional study, we extracted background information and relevant clinical data from 145 patients who were undergoing maintenance HD three times a week at the Affiliated Hospital of Nantong University between January 1998 and January 2019. The study subjects were divided into four groups according to the duration of their HD: <5 years, 5–10 years, 10–15 years, and >15 years of HD. We collected the medical history and relevant clinical parameters for each subject, and measured the urea reduction ratio (URR), hemoglobin (Hb), serum calcium, phosphorus, parathyroid hormone (iPTH), and serum albumin (ALB) levels for each group.

**Results:**

The average patient age was 52.06 ±  11.93 years old. The average patient age in the 10–15 years and >15 years groups was significantly lower than in the <5 years and 5–10 years groups (*P* = 0.002, *P* < 0.001, *P* = 0.012, and *P* = 0.0025, respectively). The most common cause of end-stage renal disease (ESRD) was chronic glomerulonephritis. We found no significant differences in URR, Hb, serum calcium, serum phosphorus, iPTH, and ALB levels.

**Conclusion:**

A prolonged HD duration was related to a younger mean age at the start of HD treatment. The leading cause of ESRD was chronic glomerulonephritis. We predominantly found diabetic nephropathy in the group with a duration of <5 years cumulative HD. Most of the indexes related to hemodialysis almost satisfied the recommended values in these patients.

## Introduction

Despite the continuously improving health care system in China, the number of patients undergoing maintenance hemodialysis (HD) is rapidly growing and the number of kidney transplant donors remains limited (Eckardt et al., 2013). HD, a relatively safe treatment for end-stage renal disease (ESRD) patients, is still widely used, and advanced technology and therapeutic strategies have improved the survival rate of HD patients (Tattersall et al., 2007; Slinin et al., 2015). Potential strategies that can reduce complications and incidence while improving prognosis and long-term survival rates in patients undergoing maintenance HD are gaining increased attention. The survival rates of patients undergoing maintenance HD depend on adequate HD performance. Inadequate HD is independently associated with increased morbidity and mortality (Chandrashekar, Ramakrishnan & Rangarajan, 2014). Following the establishment of the Kidney Disease Improving Global Outcomes (KDIGO) national and regional guidelines (European Best Practice Guidelines Expert Group on Hemodialysis ERA , 2002; Andrassy, 2013; National Kidney F, 2015; Adequate Hemodialysis Work Group of the Chinese Medical Doctor Association, 2015), Port et al. (2004) suggested that adhering to and improving certain guidelines could further prolong patient life expectancy. Specifically, adequate HD, mineral-bone metabolism, improved anemia, improved nutritional status, controlled weight gain during HD, and less frequent application of catheters for vascular access have been associated with prolonged survival (Port et al., 2004). Several studies have reported on the negative correlation between hemoglobin (Hb) levels and poor survival (Collins, 2002; Avram et al., 2003). Moreover, Owen, et al. (1993) found a 6.7-fold increase in mortality when serum albumin (ALB) levels were < 30 g/L (Owen Jr et al., 1993; Dwyer et al., 2005). However, no studies have grouped patients according to different HD durations and analyzed the clinical characteristics of each group and related HD treatment indices. This exploratory study explores the causes of ESRD and the clinical characteristics of patients undergoing maintenance HD across different cumulative durations. We hypothesized that the main cause of ESRD was chronic glomerulonephritis and that the group with a longer cumulative HD duration would adhere more to clinically relevant HD treatment target parameters.

The current study aimed to: (1) compare demographic clinical characteristics; (2) explore ESRD etiology and the incidence of concomitant diseases; and (3) investigate clinically relevant HD treatment parameters of adequacy, anemia, nutritional status, and metabolic bone disease across four groups with different HD durations (< 5 years, 5–10 years, 10–15 years, and >15 years).

## Materials and Methods

This was a cross-sectional study performed at the Affiliated Hospital of Nantong University’s HD center. The study was approved by the ethics committee of the Affiliated Hospital of Nantong University, Nantong, Jiangsu, China (#2020-K009). All patients underwent maintenance HD between January 1998 and January 2019. We obtained information from standardized forms filled out by providers when the patients started HD and confirmed their accuracy using the patient’s previous outpatient and inpatient medical history information. The inclusion criteria were: (1) age > 18 years; (2) regular HD treatment longer than three months, three times a week, and 4 h each session; and (3) signed informed consent. The following patients were excluded: (1) patients who had undergone kidney transplantation, and (2) patients who had undergone peritoneal dialysis.

We divided the study subjects into four groups according to their HD duration: < 5 years, 5–10 years, 10–15 years, and >15 years. Medical histories and relevant biochemical examination parameters, including current age, gender, age at the beginning of HD, causes of ESRD, and concomitant diseases, were collected from all patients. We calculated the urea reduction ratio (URR), which is an index of HD adequacy, as the difference between pre-dialysis blood urea nitrogen (BUN) and post-dialysis BUN, divided by pre-dialysis BUN (Liang, Zhang & Palevsky, 2019). Blood routine test, serum phosphorus, serum calcium, parathyroid hormone (iPTH), and serum ALB levels were used to evaluate the patients’ anemia management, bone mineral metabolism, and nutritional status. We compared the background information and relevant indicators of each group. The recommended values were taken from the Chinese clinical guidelines for adequate HD treatment (Adequate Hemodialysis Work Group of the Chinese Medical Doctor Association, 2015).

We used SPSS 19.0 (IBM, Armonk, NY, USA) statistical software for data processing. Measurement data with normal distribution were shown as mean ± standard deviation (SD). The multiple-group comparison was performed using an ANOVA test, while the two groups were compared using a *t*-test. Measurement data with non-normal distributions were shown as the median (M) (25th–75th interquartile range). We used a Pearson chi test to compare the categorical data between groups. A *P*-value less than 0.05 was considered statistically significant.

## Results

### Background information of maintenance HD patients

**Figure 1:**
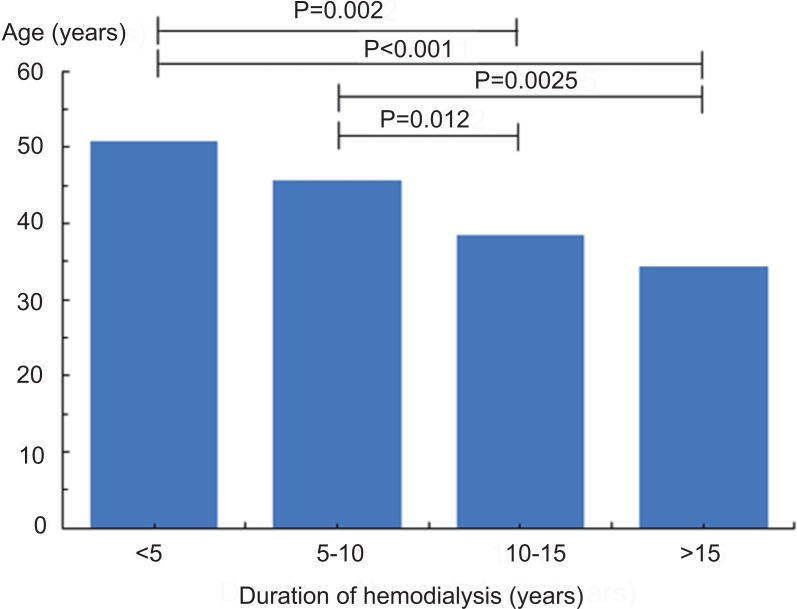
Comparison of the average age among patients starting hemodialysis in each group. As the duration of hemodialysis became longer, the average age at the beginning of hemodialysis became significantly smaller. *p* = 0.002 <5 versus 10–15, *p* < 0.001 <5 versus >15, *p* = 0.012 5–10 versus 10–15, *p* = 0.0025 5–10 versus >15.

**Figure 2:**
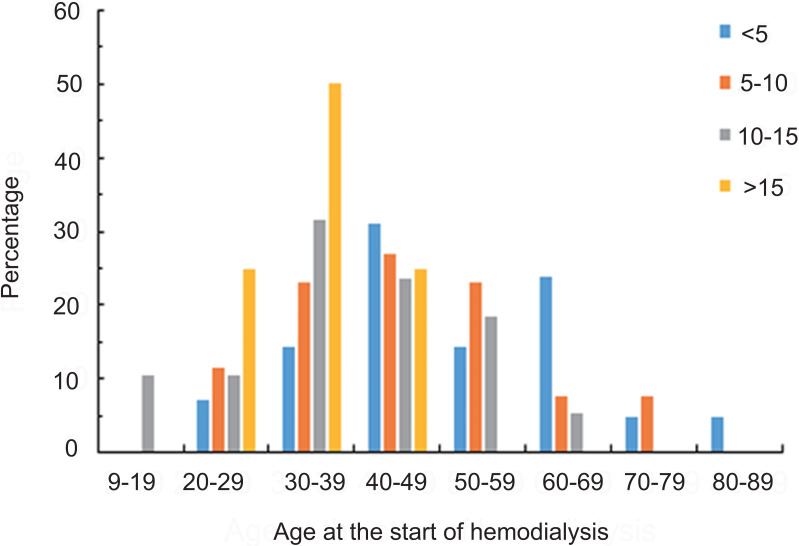
The distribution of the percentages of patients with different hemodialysis durations within each age group. Patients with hemodialysis <5 years were 25 to 82 years old at the beginning of hemodialysis. Patients with hemodialysis 5–10 years were 21 to 74 years old at the beginning of hemodialysis. Patients with hemodialysis 10–15 years were 14 to 67 years old at the beginning of hemodialysis. Patients with hemodialysis >15 years were 26 to 47 years old at the beginning of hemodialysis.

A total of 145 maintenance HD patients were enrolled in this study: 87 males and 58 females, with a 1.5:1 male to female ratio. The ages ranged from 26 to 86 years, with an average age of 52.06 ± 11.93 years. Patients were divided into four groups according to the duration of their HD treatment. There were 43 cases in the < 5 years group, 52 cases in the 5–10 years group, 38 cases in the 10–15 years group, and 12 cases in the > 15 years group. The statistical results of the patient baseline data from each group are shown in Table 1. There were no significant gender differences across the four groups. The average HD duration for the four groups was 3.93 ± 0.83 years, 7.32 ± 1.41 years, 11.54 ± 1.17 years, and 18.36 ± 1.71 years, respectively (all p <0.05). The average age of patients with an HD duration > 15 years was relatively young (49.82 ± 12.26), but it was not statistically significant. When compared with patients in the < 5 years and 5–10 year group, the 10–15 years and >15-years group had significantly lower average ages at the start of HD (all p <0.05). As the HD duration increased, the average age at the beginning of HD was significantly lower, as shown in Fig. 1. The ages at which patients from each group started HD are shown in percentages in Table 2 and Fig. 2. Patients undergoing HD > 15 years were between 26 and 47 years old at the beginning of their HD treatment.

**Table 1:** Comparison of background information, the etiology of ESRD and incidence of concomitant diseases.

	Duration of hemodialysis (years)			
	<5 (*n* = 43)	5–10 (*n* = 52)	10–15 (*n* = 38)	>15 (*n* = 12)
Male n (%)	29(67.44)	28(53.85)	22(57.89)	8(66.67)
Duration of hemodialysis (years)	3.93 ± 0.83	7.32 ± 1.41^a^	11.54 ±1.17^a^	18.36 ± 1.71^a^
Age	54.74 ± 14.48	53.04 ± 13.21	50.63 ± 7.75	49.82 ± 12.26
Age at initiation of hemodialysis	50.83 ± 14.43	45.72 ± 13.57	38.5 ± 12.57^b^	34.26 ± 7.16^b^
Cause of ESRD n (%)				
Diabetic nephropathy	8(18.6)	5(9.6)	1(2.6)	0^c^
Chronic glomerulonephritis	29(67.4)	41(78.8)	30(78.9)	11(91.7)
Polycystic kidney disease	3(7.1)	3(5.8)	3(7.9)	1(8.3)
Lupus nephritis	0	2(3.8)	2(5.3)	0
RPGN	1(2.3)	0	0	0
Uric acid nephropathy	1(2.3)	0	2(5.3)	0
Multiple myeloma related kidney disease	0	1(2.0)	0	0
Other	1(2.3)	0	0	0
Comorbidity n (%)				
Diabetes	10(23.3)	8(15.4)	1(2.6)	0^d^
Hypertension	36(83.7)	41(78.9)	31(81.6)	8(66.7)
Chronic heart failure	8(18.6)	7(13.5)	3(7.9)	1(8.3)
Cerebrovascular disease	0	1(1.9)	2(5.3)	0
Thyroid disorder	1(2.3)	0	1(2.6)	1(8.3)

**Notes.**

ESRD end stage renal disease RPGN rapidly progressive glomerulonephritis

a *p* = 0.045 <5 versus 5–10 *p* = 0.038 5–10 versus 10–15 *p* = 0.027 10–15 versus >15.

b *p* = 0.002 <5 versus 10–15 *p* < 0.001 <5 versus >15*p* = 0.012 5–10 versus 10–15 *p* = 0.0025 5–10 versus >15.

c *p* = 0.027 <5 versus >15.

d *p* = 0.0031 <5 versus >15.

### Comparing ESRD etiology and the incidence of concomitant diseases across the different groups

The main cause of ESRD across all groups was chronic glomerulonephritis. Diabetic nephropathy was the second most common cause of ESRD in the < 5 years and 5–10 years groups. The <5 year group had an 19.05% incidence of diabetic nephropathy, the >15 years group had an incidence of 0% (*p* = 0.027). The incidence of other etiologies, such as polycystic kidney disease, lupus nephritis, and rapidly progressive glomerulonephritis (RPGN), was not significantly different across the groups. Hypertension ranked first among the main concomitant diseases in each group, but its incidence was not statistically significant. Diabetes ranked second among all concomitant diseases in the <5 years and 5–10 years groups. The <5 year group had a 23.3% incidence of concomitant diabetes, the >15 years group had an incidence of 0/% (*p* = 0.0031). The incidence of other concomitant diseases, such as chronic heart failure, cerebrovascular disease, and thyroid dysfunction, was not significantly different across the four groups (Table 1).

**Table 2:** Comparison of the distribution of the percentages of patients with different HD durations within each age group.

	Duration of hemodialysis (years)			
Age at initiation of hemodialysis	<5 n(%)	5–10 n(%)	10–15 n(%)	>15 n(%)
9–19 20–29 30–39 40–49 50–59 60–69 70–79 80–89	0 3(6.98) 6(13.95) 14(32.56) 6(13.95) 10(23.26) 2(4.65) 2(4.65)	0 6(11.54) 12(23.08) 14(26.92) 12(23.08) 4(7.69) 4(7.69) 0	4(10.53) 4(10.53) 12(31.58) 9(23.68) 7(18.42) 2(5.26) 0 0	0 3(25.00) 6(50.00) 3(25.00) 0 0 0 0

### Comparing relevant HD treatment parameters across different groups

The URR, Hb, serum calcium, and ALB levels across the groups were consistent with the recommended guidelines (Adequate Hemodialysis Work Group of the Chinese Medical Doctor Association, 2015). The <5 years group and the 10–15 years group had iPTH values that were within the recommended values, while the 5–10 years group and the >15 years group had iPTH values that were higher than the recommended values. The serum phosphorus values across the groups were also higher than the recommended values. The URR, Hb, serum calcium, serum phosphorus, iPTH, and ALB levels were not significantly different across the four groups (Table 3).

**Table 3:** Comparison of hemodialysis-related parameters in patients undergoing maintenance hemodialysis for different years.

Clinical parameters	Duration of hemodialysis (years)				*p*-value	Recommended values^a^
	<5	5–10	10–15	>15		
URR(%)	66.23 ± 6.68	68.33 ± 6.05	69.1 ± 4.81	66.4 ± 7.16	0.68	≥ 65
Hb(g/L)	102.29 ± 12.58	100.81 ± 10.32	104.42 ± 19.6	108.38 ± 10.72	0.72	≥100
Calcium (mmol/L)	2.17 ± 0.25	2.29 ± 0.42	2.32 ± 0.23	2.18 ± 0.32	0.56	2.1-2.75
Phosphorus(mmol/L)	1.81 ± 0.43	1.97 ± 0.51	1.92 ± 0.58	2.01 ± 0.52	0.22	1.13-1.78
iPTH(pg/ml)	291.29 ± 466.92	304.13 ± 463.76	299.66 ± 482.95	311.74 ± 413.7	0.43	150-300
ALB(g/L)	40.76 ± 2.61	40.53 ± 2.18	40.62 ± 1.84	39.74 ± 1.72	0.65	≥35

**Notes.**

a Recommendation values were followed by Chinese clinical guidelines for adequacy of hemodialysis.

URR Urea reduction ratio Hb Hemoglobin iPTH intact parathyroid hormone ALB Albumin;1g/L = 0.1 g/dL; Calcium (1 mmol/L = 4.008 mg/dL); Phosphorus(1 mmol/L = 3.0969 mg/dL)

## Discussion

For over 50 years, HD has continued to improve, making it a very popular and commonly-used treatment. In the past, HD was generally connected to poor prognosis (Zazzeroni et al., 2017), which is why the long-term survival rate of HD did not receive much attention from the scientific community. Few studies have reported on the factors that can increase the survival rate for HD patients. In this study, we examined the clinical characteristics of patients undergoing maintenance HD for <5-years, 5–10 years, 10–15 years, and >15 years, and we found a correlation between a significantly younger age at the beginning of HD and a relatively longer HD duration. The leading cause of ESRD was chronic glomerulonephritis. In order to improve the prognosis of patients with diabetic nephropathy, compliance with HD standards during maintenance HD treatment should be emphasized.

Our results showed that a longer cumulative HD duration was related to a younger mean age at the start of HD treatment. Patients undergoing maintenance HD for over 15 years had an average starting age of 34.26 ± 7.16 years, suggesting that patients who started HD at a younger age underwent longer maintenance HD treatment, which is consistent with previous reports (Heaf, Nielsen & Hansen, 2012). The age at which HD was initiated may be a prognosis factor for patients undergoing maintenance HD for over 15 years. Additionally, patients who started HD at a younger age will remain on HD for a longer duration unless they die prematurely. This may also explain why the group with a longer cumulative HD duration had more cases of chronic glomerulonephritis, as chronic glomerulonephritis tends to present in younger age groups and lead to rapid ESRD. Diabetes mellitus type 2 (DM2) tends to cause ESRD only after being present for many years in older patients. In this study, the leading cause of ESRD in HD patients was chronic glomerulonephritis. The percentage of patients with chronic glomerulonephritis across the groups was 66.67%, 79.25%, 78.95%, and 91.67%, respectively. Diabetic nephropathy was the second most common cause in the <5 years and 5–10 years groups. Notably, the incidence of diabetic nephropathy causing chronic renal failure was 0% in the >15 years group, which suggests that there were no diabetic patients in this group. This was in line with the results reported by Otsubo et al. (2007), who found that the prognosis of renal failure secondary to diabetes was worse than other ESRD causes. The prognosis for patients with diabetic nephropathy needs to be examined and improved.

Receiving adequate HD treatment is a key factor in the overall management of HD patients. In this study, we observed similar URRs across all groups. All groups successfully achieved the limits recommended by the guidelines, which indicated that all subjects underwent adequate HD treatments. In order to decrease morbidity and mortality, the following factors should be properly managed in populations that are dependent on maintenance HD: anemia, bone-mineral metabolism, and nutritional status (Port et al., 2004). The National Kidney Foundation’s Kidney Disease Outcomes Quality Initiative (KDOQI) and other relevant Chinese guidelines all recommend that HD patients should maintain Hb levels of 100 g/L, and iron-deficient patients should use iron and erythropoietin to achieve this level (National Kidney F, 2015; Adequate Hemodialysis Work Group of the Chinese Medical Doctor Association, 2015). Some retrospective studies recommended a serum Hb level of 10 g/dL to 11 g/dL (Mikhail et al., 2017; Tanaka, Komaba & Fukagawa, 2018). Serum Alb levels are an effective and clinically useful indicator of protein-energy nutritional status in patients undergoing maintenance HD (Sabatino et al., 2018). We observed a strong negative correlation between HD patients’ mortality and serum Alb levels (Roach et al., 2017), which should be maintained at 35 g/L (National Kidney F, 2015; Adequate Hemodialysis Work Group of the Chinese Medical Doctor Association, 2015). Patients with serum Alb concentrations below 35 g/L have a 1.5 times higher risk of death (Roach et al., 2017).

In this study, serum Alb levels were a satisfactory indicator of the patients’ nutritional status, and each group met the recommended standards. In ESRD patients, changes in bone-mineral metabolism generally lead to soft tissue and blood vessel calcification (Neves et al., 2017). According to Young et al.(2005), Block et al. (2004), these changes may lead to blood vessel occlusion in the coronary arteries and peripheral and cerebral circulation, which can ultimately increase morbidity and mortality rates. Although KDIGO recommends that the target iPTH levels in HD patients should be two to nine times greater than the upper limit of the standard reference value, multiple clinical studies have shown that patients with iPTH levels between 150 and 300 pg/ml have lower mortality rates (Floege et al., 2011; Naves-Diaz et al., 2011; Kalantar-Zadeh et al., 2010). In this study, the patients had standard serum calcium levels. However, the iPTH levels were higher than the recommended values in the 5–10 years and >15 years groups, and the serum phosphorus levels exceeded the recommended values in each group. A previous study found a correlation between the mortality of HD patients and their serum phosphorus concentrations (Ma, Zhao & Li, 2017). Additionally, the mortality rate of patients with low phosphorus concentrations also increased, which may have been due to nutritional deficiencies (Ma & Zhao, 2017). Patients with increased serum phosphorus levels should therefore have stricter dietary guidance, properly adjusted drug regimens and HD programs, and improved monitoring.

There were some limitations in this study. First, this was a single-center cross-sectional study with a small sample size. Second, when measuring the adequacy of the HD treatment, we did not measure single pool Kt/V (spKt/V) values, which is a more accurate method. Third, we failed to investigate which dialysis techniques and membranes could most impact survival. Future studies should expand the sample size, focus on patients undergoing HD for more than 15 (or even as long as 30) years, conduct multivariate analyses with more parameters, and more comprehensively assess the patients’ quality of life.

## Conclusion

In this study, we investigated the clinical characteristics of patients with different cumulative HD durations and emphasized the importance of adherence to clinical guidelines for these patients. Chronic glomerulonephritis was the leading cause of ESRD, followed by diabetes mellitus. As HD durations increased, the percentage of renal failure caused by diabetic nephropathy decreased. There were no patients with diabetic nephropathy in the > 15 years group, suggesting that patients with diabetic nephropathy either survived for shorter periods on HD or that patients had an increased mortality rate because they started HD at a later age. It is essential to improve the prognosis of diabetic nephropathy patients who develop ESRD. Most of the patients satisfied the recommended values for the HD treatment indices, with the exception of phosphorus and iPTH levels. This suggests that greater attention should be paid to managing patient metabolic bone disease parameters, regardless of their cumulative HD duration.

##  Supplemental Information

10.7717/peerj.10852/supp-1 Supplemental Information 1 Raw data.

## Additional Information and Declarations

### Data Availability

The following information was supplied regarding data availability: Raw measurements are available as a Supplementary File.
